# Genetic Diversity of *Blumeria graminis* f. sp. *hordei* in Central Europe and Its Comparison with Australian Population

**DOI:** 10.1371/journal.pone.0167099

**Published:** 2016-11-22

**Authors:** Eva Komínková, Antonín Dreiseitl, Eva Malečková, Jaroslav Doležel, Miroslav Valárik

**Affiliations:** 1 Institute of Experimental Botany, Centre of the Region Haná for Biotechnological and Agricultural Research, Olomouc, Czech Republic; 2 Department of Integrated Plant Protection, Agrotest Fyto Ltd., Kroměříž, Czech Republic; GERMANY

## Abstract

Population surveys of *Blumeria graminis* f. sp. *hordei* (*Bgh*), a causal agent of more than 50% of barley fungal infections in the Czech Republic, have been traditionally based on virulence tests, at times supplemented with non-specific Restriction fragment length polymorphism or Random amplified polymorphic DNA markers. A genomic sequence of *Bgh*, which has become available recently, enables identification of potential markers suitable for population genetics studies. Two major strategies relying on transposable elements and microsatellites were employed in this work to develop a set of Repeat junction markers, Single sequence repeat and Single nucleotide polymorphism markers. A resolution power of the new panel of markers comprising 33 polymorphisms was demonstrated by a phylogenetic analysis of 158 *Bgh* isolates. A core set of 97 Czech isolates was compared to a set 50 Australian isolates on the background of 11 diverse isolates collected throughout the world. 73.2% of Czech isolates were found to be genetically unique. An extreme diversity of this collection was in strong contrast with the uniformity of the Australian one. This work paves the way for studies of population structure and dynamics based on genetic variability among different *Bgh* isolates originating from geographically limited regions.

## Introduction

Since the onset of agriculture, cereals have played a crucial role in human nutrition. However, the needs of the growing human population, which is projected to reach 9.7 billion by 2050 [1], together with the changing climate represent a serious challenge for breeders and researchers to meet the growing demand for food. Despite the fact that barley represents only 5.2% of the total world production of cereals with 144 mil. tons harvested per year [2], this cereal ranks among major crops in many, especially European countries responsible for 59.7% of total world production. The Czech Republic has a long tradition in barley production mainly due to the brewery industry with 14.9% of sowing areas occupied by this crop and its yearly harvest representing as much as 22.4% of total cereal production [3].

The yield is constantly exposed to the risk of adverse effects due to different abiotic and biotic factors. An obligate biotrophic fungus *Blumeria graminis*, a causal agent of powdery mildew disease, is currently ranked among the Top 10 most important fungal plant pathogens [4]. *B*. *graminis* f. sp. *hordei* (*Bgh*) is responsible for 57% and 51% of leaf disease epidemics occurring on spring and winter barley, respectively, in local conditions [5–6]. To prevent yield losses, breeding resistant cultivars offers an effective crop protection strategy without the need for fungicides. However, interaction between host and pathogen is a highly intricate and dynamic process and our understanding of its complexity is a necessary prerequisite to reach the goal.

To study the pathogen diversity and populations, phenotypic traits including virulence and sensitivity to different fungicides used to be the only approach available before the rise of molecular biology [7–19]. However, in spite of providing valuable data, such an analysis itself can be limiting and its combination with genotype data is therefore highly desirable.

Initially, markers based on isozymes were used in addition to phenotype data. While they proved to be sufficient to characterize genetic variation between different *formae speciales* (ff. spp.) of *B*. *graminis*, they showed very low level of polymorphism within individual ff. spp. [20,21]. Later on, the first generation of DNA markers provided a widely-used and relatively efficient tool for characterizing *Bgh* populations in addition to virulence gene studies [22–25]. Considerably low genetic variation resulting in majority of isolates sharing the same Restriction Fragment Length Polymorphism (RFLP) pattern in some cases was explained by their clonal origin [22] or geographical isolation together with a lack of selection pressure [25]. In contrast, a number of studies [26–28] reporting application of non-specific Random Amplified Polymorphic DNA (RAPD) markers on *Bgh* isolates collected either across Europe or on single localities indicated a large genetic variation, even within common pathotypes. More recently, Single Nucleotide Polymorphism (SNP) markers were applied to study evolutionary relationships between different ff. spp. of *Bgh*. Wyand and Brown [29] investigated polymorphism of β-tubulin gene and ITS regions both of which did not show any variation within any f. sp., whereas Inuma *et al*. [30] relied additionally on chitin synthase 1 gene and 28S rDNA. Oberhaensli *et al*. [31] were the first to use polymorphism in non-coding intergenic sequence including transposable elements (TEs). However, variation among isolates of single f. sp. has not been reported.

Until recently, Single Sequence Repeat (SSR) markers were rarely used in *B*. *graminis* due to time-consuming and laborious development. Wang *et al*. [32] described development of SSR markers suitable for analysis of genetic diversity between isolates of *B*. *graminis* f. sp. *tritici* (*Bgt*) from microsatellite-enriched genomic libraries. A crucial milestone in *Bgh* research have been achieved by whole genome sequencing of strain DH14 performed by Spanu *et al*. [33]. The analysis of genome sequence revealed massive colonization by TEs, predominantly retrotransposons, accounting for 64% of the genome size. Whole genome sequencing of two additional *Bgh* isolates and their comparative analysis with the reference genome DH14 revealed highly polymorphic isolate-specific DNA blocks indicating large genetic variation in the *Bgh* population [34]. Tucker *et al*. [35] were the first to employ the genome sequence data for developing SSR markers to characterize a set of Australian *Bgh* isolates. Together with the possibility of developing markers based on TEs or SNPs, the genomic sequence provides a rich source of polymorphism for diversity and population studies.

In our preliminary experiments, we examined the possibility to use ITS sequences supplemented with markers derived from glyceraldehyde-3-phosphate dehydrogenase gene for assessment of *Bgh* genetic diversity. However, no polymorphism was detected among 14 tested *Bgh* isolates.

A study comparing phenotypes of Central European and Australian isolates reported large difference in virulence complexity between the two populations [36]. This finding raises a question whether the observed diversity in phenotype corresponds to genetic variability. Considering the geographically limited area of the Czech Republic, there are two possible population structure hypotheses. The first one suggests high variability of *Bgh* isolates with different genotypes, the second one anticipates rather small number of widespread genotypes. Clonal propagation and airborne spread of the pathogen during growing season together with low variability in sequences of “housekeeping” genes and ITS support the hypothesis of low number of genotypes. In contrast, presence of isolates with significantly higher virulence complexity in comparison to Australian *Bgh* population prefers the hypothesis of high genetic variability among *Bgh* isolates facilitated by evolutionary forces favored in Central European conditions (high gene flow, population size and selection pressure exerted by deployment of different R genes in grown barley cultivars). To resolve the question, we mined the DH14 genomic sequence [33] to find microsatellites and retrotransposons suitable for designing new markers. The main objective of this study was i) development of a marker panel with sufficient resolution within the population of Czech *Bgh* isolates and ii) comparison of genetic diversity between Czech and Australian populations.

## Results

### Marker development

Repeat Junction Markers (RJM) were designed manually from retrotransposons of superfamilies *Copia* and *Gypsy*. 10 contigs containing complete *Copia* or *Gypsy* element including Long Terminal Repeats (LTRs) and Target Site Duplication (TSD) delimiting the retrotransposon insertion site were selected. Out of 20 RJM-derived primer pairs (termed *obm1-obm20*) (S1 Table), five revealed presence/absence variation (PAV) (*obm13*, *obm14*, *obm15*, *obm16*, *obm18*). However, they belonged only to three different insertion sites and as a result, two pairs of markers provided redundant information (*obm13*/*obm14* and *obm15*/*obm16*). Additionally, *obm15*/*obm16* failed to amplify reproducibly and thus, only markers *obm14* and *obm18* were considered.

Eleven primer pairs (*obm2*, *obm3*, *obm4*, *obm6*, *obm7*, *obm8*, *obm9*, *obm10*, *obm17*, *obm19*, *obm20*) provided single fragment of expected size and were selected as candidates for sequencing and SNP discovery. Remaining primer pairs produced multiple PCR products (*obm1*) or no product at all (*obm5*, *obm11*, *obm12*). Six of the sequenced amplicons (*obm2*, *obm3*, *obm4*, *obm7*, *obm8*, *obm19*) showed 100% identity across all testing isolates and were not used in further work. *Obm17* was discarded as well because it yielded a mixture of amplicons of the same length. Primer pairs *obm9* and *obm10* provided SNPs with redundant genotypes and thus *obm9* was considered only.

In summary, this strategy resulted in identification of two reliable PAV markers (*obm14*, *obm18*) and three PCR products (*obm6*, *obm9*, *obm20*) suitable for SNP development (S1 Table). Each identified SNP was scored as individual polymorphism. The *obm6* amplicon yielded four SNPs which were marked as *obm6*.*1*—*obm6*.*4*. The *obm9* yielded two SNP markers designated as *obm9*.*1* and *obm9*.*3*. (marker *obm9*.*2* showed 63 bp indel detected only in isolates Y-069 and H-148 originating from Israel). Finally, *obm20* yielded seven SNP markers, *obm20*.*1* –*obm20*.*7* (S2 Table). SNP marker designated *obm20*.*6* was omitted from further analysis since it provided identical genotypes as *obm20*.*2*.

Out of 10 SSR-based primer pairs (*obm21*-*obm30*) derived from random DH14 sequence scaffolds, four (*obm24*, *obm27*, *obm28*, *obm29*) produced five, three, three and seven polymorphic bands, respectively. Two primer pairs, *obm22* and *obm26*, produced too complex patterns (*obm22* yielded as much as 48 length variants) for reliable scoring and the markers were not used further. Remaining four primer pairs provided monomorphic amplicons (S3 Table). Different length variants of *obm24*, *obm27*, *obm28* and *obm29* were scored separately and provided sixteen reliable polymorphisms labeled in the same manner as the SNP markers.

### Genotyping of *Blumeria graminis* isolates

50 isolates collected in Australia (AUS), a subset of 11 isolates (W) originating from the genebank maintained at Agrofest Fyto Ltd. and 97 isolates (CZE) collected in the main barley growing regions of the Czech Republic were genotyped using the panel of markers described above. Altogether, 33 polymorphisms were scored and 5,214 data points were obtained. Among all tested isolates, each SNP marker provided two variants. All detected polymorphisms including PAV in RJMs and SSR markers or different variants of nucleotides in SNP markers could be therefore converted to binary data 0/1 or “?” in case of missing or unreliable genotypes. The missing data were observed in 17 cases and represented only 0.33% of the whole dataset. The highest number of polymorphisms (28) was observed among Czech isolates. Only five markers (*obm9*.*2*, *obm24*.*1*, *obm28*.*3*, *obm29*.*3*, *obm29*.*5*) were monomorphic. On the other hand, polymorphism was detected only by seven markers for Australian isolates (*obm6*.*1*, *obm6*.*2*, *obm9*.*3*, *obm24*.*3*, *obm24*.*4*, *obm29*.*2*, *obm29*.*6*, *obm29*.*7*). Finally, 24 polymorphic markers were observed within the subset of world collection of *Bgh* and nine markers were monomorphic (*obm6*.*3*, *obm6*.*4*, *obm20*.*1*, *obm20*.*3*, *obm20*.*5*, *obm27*.*1*, *obm27*.*3*, *obm29*.*1*, *obm29*.*7)*. The W set comprised five private polymorphisms while the CZE eight and the AUS did not show any private polymorphism.

### Pathogen diversity

**Fig 1:**
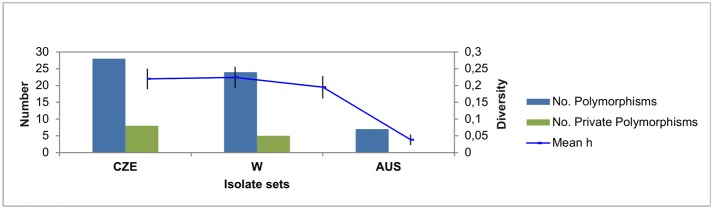
Polymorphism patterns across sets of *Bgh* isolates inferred from genotyping by SSR, RJM, indel and SNP markers. The number of polymorphisms observed in the population of isolates collected in the Czech Republic in season 2012 (CZE), collection of isolates from around the world (W) and population of isolates collected around Australia in season 2011 (AUS) is represented by the blue column. The green column illustrates amount of private polymorphisms. The mean diversity (Mean h) among the sets of isolates is marked by blue line and vertical bars represent standard error.

Molecular variance among and within isolate sets and its polymorphism patterns are summarized in the Table 1 and Fig 1.

**Table 1:** Analysis of molecular variance (AMOVA) for 172 individuals from 4 *Bgh* isolate sets based on 33 polymorphisms derived from SSR, RJM, indel and SNP markers.

*Source of variation*	*Degrees of freedom*	*Sum of squares*	*Means square*	*Est*. *Var*.	*% variation*	*P value*
Among Pops	3	104.684	34.895	0.954	25%	0.001
Within Pops	168	469.401	2.794	2.794	75%	0.001
Total	171	574.085		3.748	100%	0.001
ΦPT = 0.255						0.001

The W set has the highest diversity (0.224) comparable with the CZE set (0.220) and the lowest diversity (0,038) was observed in the A set (Fig 1). Results of the AMOVA (Table 1) indicated that most (75%) of the molecular variation is present among individuals within populations whereas only 25% was detected among populations. Permutation tests (based on 999 permutations) suggest that the overall ΦPT was significant (ΦPT = 0.255, P = 0.001; Table 1) which indicates that the differences among the sets are significant. Pairwise population ΦPT analysis identified relatively high ΦPT 0.341 and 0.349 from comparison of CZE and W with AUS set, respectively. On the other hand, the ΦPT from comparison of the CZE and W set was only 0.084.

**Fig 2:**
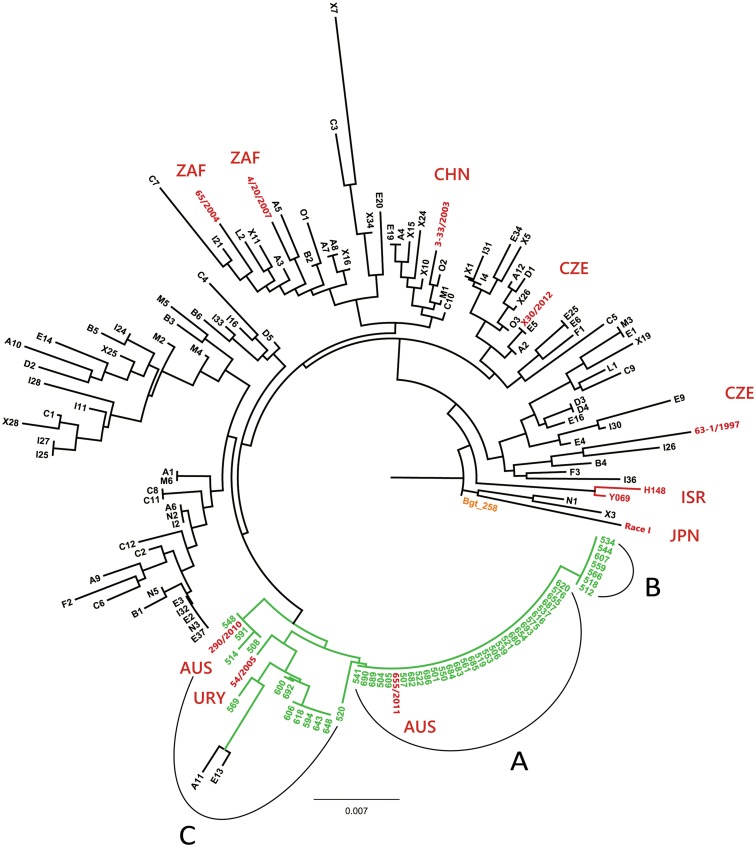
Resolution of the marker panel demonstrated using phylogenetic tree. Phylogenetic tree of *Blumeria graminis* f. sp. *hordei* (*Bgh*) isolates based on 33 polymorphisms (RJMs, SSRs, SNPs). The outgroup (*Bgt*) isolate *Bgt258* is highlighted in orange, Australian isolates collected in 2011 are marked green, isolates of the worldwide collection (CHN = China, CZE = Czech Republic, ISR = Israel, JPN = Japan, URY = Uruguay, ZAF = South African Republic) are highlighted in red and the remaining isolates in black originate from collection made in the Czech Republic in 2012. Letters A, B and C delimit three sections of highly similar Australian isolates. Please note that only few Czech isolated remained undistinguished proving high resolution power of the marker panel. Interestingly, the Czech isolates show no association with area of collection (Fig 3). Additionally, samples representing the worldwide *Bgh* diversity are evenly distributed within the Czech isolates. The exception is isolate Race I originating from Japan and collected more than 60 years ago and two isolates (H148, Y069) collected in Israel. On the other hand, the Australian isolates show low genetic variability and wide spreading of the observed haplotypes. The A) section represents *Bgh* haplotype spread through whole Australian barley growing areas (Fig 4). The B) section shows closely related isolates to A and found only in south east territories. The C) section represents isolates collected mostly around costal territories with high diversity level suggesting recent import from abroad. This hypothesis is supported by haplotype similarity with isolate from Uruguay.

A phylogenetic analysis of the Czech, Australian and worldwide collection of *Bgh* isolates was used to assess resolution of the marker panel and resulted in a tree (Fig 2) where isolate of *B*. *graminis* f. sp. *tritici* (*Bgt*258) was used as an outgroup. As expected, Australian isolates showed low level of diversity and majority of them (30 out of 50) shared identical genotype profiles (Fig 2, Section A). This cluster represents one isolate or highly similar family collected from all Australian barley growing regions. Another section (Fig 2, Section B) comprised 14% of isolates highly similar to those in Section A but distributed on seven localities of the southeastern Australian territories including Tasmania. The remaining, genetically more diverse isolates (Fig 2, Section C) are genetically close to the Czech and Uruguayan *Bgh* isolates.

On the other hand, majority of isolates collected within the Czech Republic (71 out of 97) constituted a separate branch providing an evidence for high resolution of developed marker panel. The exceptions were one group of five and one group of three undistinguished isolates (E2, E3, E37, I32, N3 and A6, I2, N2, respectively) together with nine pairs of isolates with identical genotype profiles. Nevertheless, no correlation between phylogenetic relationships and geographical origin of the isolates was apparent.

Among the *Bgh* isolates of the worldwide collection, the most distinct one was isolate Race I of Japanese origin collected in 1953 together with both Israeli isolates (H-148, Y-069). The remaining isolates are relatively evenly distributed across the tree with no distinct pattern (Fig 2).

## Discussion

*B*. *graminis* is able to reproduce both sexually and clonally which allows evolution of new pathotypes followed by their fast spread across host. Clarifying the host-pathogen interactions and monitoring of fungicide resistance could significantly enhance the efficiency of disease control. This task requires knowledge of the pathogen population structure and its changes in time and space including its proper molecular characterization. So far, a majority of studies on characterization of *Bgh* populations were based on RFLP and RAPD markers [22–28]. Specific, gene-based SNP markers were only applied to distinguish different *formae speciales* of *B*. *graminis* [29,30]. However, as mentioned above, our pilot study with this type of markers did not reveal polymorphism among Czech *Bgh* isolates (unpublished data). Since the gene-coding sequences did not yield any polymorphism, we focused on repetitive sequences which accumulate mutations at higher rate. While a successful development of SSR markers for Chinese *Bgt* and Australian *Bgh* isolates has been reported [32,35], markers based on TEs have not been employed so far. Our study is the first to propose them as suitable candidates for population genetic studies focused on this pathogen.

We obtained two unique and reliable PAV markers out of 20 primer pairs designed for 10 different TE insertion sites (S1 Table). Low polymorphism could be explained by recent differentiation of *Bgh* isolates resulting in low frequency of novel retrotransposon insertions. This explanation is supported by finding of Oberhaensli *et al*. [31] who reported large difference in TE content between two different *formae speciales* of *B*. *graminis* suggesting numerous TE-insertion events which occurred after their divergence dated about 10 million years ago. Nevertheless, high quality reference sequences of both *Bgh* and *Bgt* and higher number of TE-based markers would be required to make a reliable conclusion on this matter. The PAV markers were supplemented with TE-based SNPs identified by sequencing monomorphic amplicons. This strategy yielded 13 informative SNPs and one indel (S2 Table) and indicated that described approach provides a rich source of polymorphisms in intergenic regions of *Bgh* genome. Recently, SNP markers has become very popular due to their abundance and availability of high-throughput genotyping platforms [37–39]. However, such platforms require large arrays of SNP markers which are not available for *B*. *graminis* yet.

The second approach presented here benefits from abundant microsatellite sequences. Out of 10 primer pairs, six resulted in polymorphic bands representing 60% success rate. However, only four of them (*obm24*, *obm27*, *obm28*, *obm29)* could be reliably scored and provided a total of 18 alleles (S3 Table). The remaining two markers produced too many amplicons with unreproducible sizes even using fluorescent labeling and capillary fragment analysis (data not shown). A similar approach of SSR marker design and capillary fragment analysis was employed by Tucker *et al*. [35] who tested eight microsatellite loci out of 30 SSR amplicons. All of them were reported to show polymorphism and an average of seven alleles per locus was detected when genotyping a set of 111 *Bgh* isolates. In contrast, Wang *et al*. [32] did not have genome reference sequence at hand and relied on *de novo* microsatellite isolation by cloning-based approach. Analysis of 90 *Bgt* isolates resulted in five polymorphic microsatellites out of 31 tested with the mean number of observed alleles reaching 5.8. The current work together with studies mentioned above demonstrates that microsatellites offer a rich and not fully explored source of polymorphism for marker development in *B*. *graminis*.

Genotyping of analyzed samples resulted in detection of 33 polymorphisms. The dataset was subjected to AMOVA analysis which allows for a partitioning of molecular variance within and among populations and tests variance components significance using permutation test [40]. The ΦPT values, which are analogous to the fixation index Fst and are more suitable for binary-haploid dataset used in this study [41], were calculated. The ΦPT = 0.255 (P = 0.0001) for whole dataset indicated that majority (75%) of the molecular variation in the *Bgh* sets occurs among individuals within populations, with only 25% of total variation found among populations. Such high variability within populations compared to variability among populations was observed before in fungi with mixed reproductive system [42] or plants [43]. Assessment of the pairwise population ΦPT values indicates relatively high variability (34.1%) between Australian and Czech populations. Similarly, variability between the AUS population and the set of worldwide isolates was 34.9%. In contrast, comparison between CZE and W indicates only 8.4% variability between the collections. This suggests restriction in gene flow between the Australian *Bgh* population and the rest of the world. On the contrary, gene flow between the Czech *Bgh* pathotypes and pathotypes of surrounding world seems to be unobstructed. However, the W collection do not represents distinct population but rather representation of worldwide diversity among the *Bgh* pathotypes. Moreover, its small size (11 isolates) brings high level of bias and thus the results from comparisons with the W set should be considered only as suggestive. Even though the W collection is small, it provided proportionally high number of private polymorphisms and with increased number of tested pathotypes, higher diversity can be expected even compared to the CZE population.

To assess and graphically visualize the resolution power of the marker panel, the obtained data were used to construct a phylogenetic tree. The resolution power was demonstrated on a set of isolates originating from the Czech Republic among which 73.2% could be unambiguously identified (Fig 2). The level of diversity within the Czech *Bgh* population (84.5% of unique genotypes) is in agreement with phenotype survey [44] which revealed 95% of isolates with distinct virulence spectra out of 521 isolates collected between years 2011–2014. However, nine pairs of isolates from our dataset shared identical genotype profiles (Fig 2). Out of them, five pairs were collected in the same region but their phenotype profiles [44] were different with the exception of isolates I25 and I27 which might be considered redundant. Similarly, one group of three and one group of five genotypically identical isolates were identified. In the latter, another pair of isolates sharing the locality of origin together with genotype and phenotype profile was detected (E2 and E3). No additional redundancies were observed. The resulting tree topology indicates that the major characteristic of Czech isolates is a lack of correlation between their genotype and geographical origin. This phenomenon can be explained by an ability of long-distance migration of this airborne pathogen [45].

High pathotype diversity of Czech *Bgh* population contrasts with the uniformity of Australian pathotypes [36], which were also characterized by low DNA polymorphism, exhibited low resolution in the phylogenetic tree and formed relatively compact clusters (Fig 2). The low diversity of Australian population is supported by pathogenicity survey of 362 Australian isolates including those analyzed in the present study [46]. Virulence assessment using 32 differential barley varieties resulted in detection of mere 27 different pathotypes with 92% of all isolates belonging to 15 of them. Low level of both genetic and phenotypic variation within Australian *Bgh* population was described earlier. Among 57 isolates collected in different regions of Australia, Whisson [25] obtained identical pathogenicity profiles using 22 differential barley lines and no polymorphism was detected by RFLP analysis using a probe that had previously provided variable fingerprints within British *Bgh* population. Limited virulence and genotype variability of the Australian *Bgh* population is in concordance with its isolation from the rest of the world which restricts gene flow [11,36]. Moreover, the breeding practices in Australia and Europe have been in strong contrast. Europe has long history of barley production with wide range of host varieties (of both spring and winter barley) containing many specific resistance genes. Such long-term directional selection posed upon the pathogen is the major factor increasing virulence complexity of Central European pathotypes. On the other hand, employment of specific resistances has been so far negligible in barley cultivars grown in Australia. This substantial difference makes the two studied populations real extremes.

On the contrary, Tucker *et al*. [35] reported high level of genetic diversity using eight SSR markers within a collection of 111 Australian *Bgh* isolates which constituted 97 unique haplotypes. To cast more light on the discrepancy, we tested all eight SSR markers of Tucker *et al*. [35] on our set of Australian isolates. In one case, no PCR product was obtained. Another two markers were monomorphic after separation by polyacrylamide gel electrophoresis. One marker yielded too complex pattern to be reproducibly scored while the remaining four markers showed polymorphism and provided new data to extend our results.

This work describes an effective strategy for development of markers for population genetics-based studies of cereal powdery mildew pathogen. The markers can be reliably scored using gel electrophoresis and easily converted for use by high-throughput genotyping systems. With only nine primer pairs we identified 33 polymorphisms useful as markers suitable for molecular characterization of *Bgh* isolates from the Czech Republic. 5,214 acquired data points and comparison of the Czech and Australian *Bgh* populations suggests that a higher genetic variability is linked with a higher relative virulence complexity. Additional knowledge on population structure and eventually dynamics might contribute to effective control measures and pathogen-informed strategy for sustainable and broad-spectrum crop resistance.

## Materials and Methods

### *Blumeria graminis* isolates

**Fig 3:**
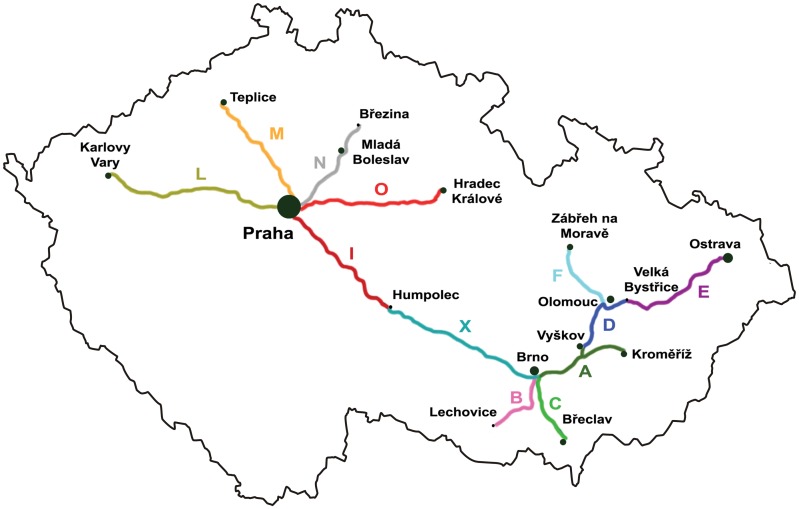
Localities of *Blumeria graminis* f. sp. *hordei* collection in the Czech Republic. The country is divided into sub-regions corresponding to highways passing through main barley growing areas. Each sub-region is labeled as color line and marked with different letter. The letters corresponds to isolate designations in S5 Table. Numbers of isolates collected in individual sub-regions are as follows: A (12), B (6), C (12), D (5), E (15), F (3), I (15), L (2), M (6), N (4), O (3), X (14).

**Fig 4:**
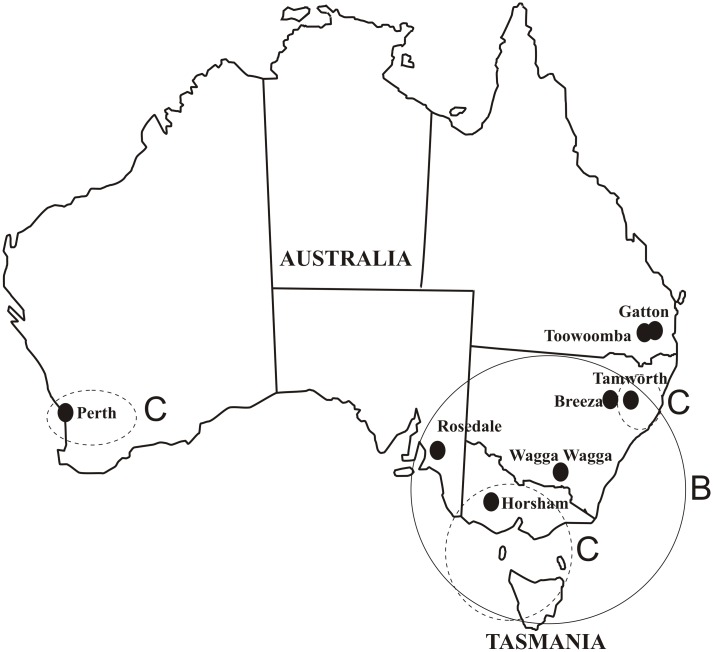
Origin of Australian *Blumeria graminis* f. sp. *hordei* isolates. Number of isolates collected on individual localities is as follows: Perth (5), Rosedale (6), Horsham (5), Tasmania (7), Wagga Wagga (9), Tamworth (10), Toowoomba (6), Gatton (2). Since the Australian isolates exhibit low level of genetic variability, only three distinct groups were identified (Fig 2). The major haplotype A) is spread through whole Australia and may represent the oldest *Bgh* introduction. The haplotype B) which is highly similar to A) and found only in the south east territories. C) The group with relatively high diversity and similarity to abroad isolate from Uruguay was found only in the costal territories and may represent the latest influx of *Bgh* isolates into Australia.

Four sets of *Blumeria graminis* (DC.) Golovin ex Speer f. sp. *hordei* Em. Marchal isolates were used for marker development, validation and diversity assessment. The first one represented fifteen isolates selected for marker testing. This group consisted of twelve isolates from 2009 collection around the Czech Republic [47], two isolates from the South African Republic [48] and one isolate of *Bgt* (*Bgt*258) collected in Olomouc, Czech Republic in 2010 which was used as an outgroup (S7 Table). The second set comprised a collection of 97 isolates originating from the main barley growing areas of the Czech Republic collected in season 2012 [44] (S5 Table, Fig 3). The third set included 50 isolates from barley growing areas of Australia collected in 2011 [46] (S6 Table, Fig 4). The fourth set was a selection of 11 reference isolates (S7 Table) of the pathogen genebank built as a core collection at the Agrotest Fyto Ltd. The genebank comprises isolates collected around the world during the past six decades and includes also Israeli isolates collected on wild barley (*Hordeum vulgare* subsp. *spontaneum*) [49].

### DNA extraction

About 10–20 mg of spores were enzymatically treated for 2 hours at 37°C in 150 μl solution of 10 mg·ml^-1^ Lysing Enzymes from *Trichoderma harzianum* (syn. Glucanex^®^, Sigma-Aldrich, USA), 1% Triton X-100 (Sigma-Aldrich, USA), 4% 2-mercaptoethanol (Sigma-Aldrich, USA), and 50 mmol·l^-1^ EDTA (Sigma-Aldrich, USA), pH 5.6. Samples were stirred several times during the treatment. Subsequently, 100 μl of lysis solution consisting of 500 mmol·l^-1^ NaCl, 100 mmol l^-1^ Tris-HCl, 50 mmol·l^-1^ EDTA (pH 8.0), 0.02% Sodium Dodecyl Sulphate, (Serva, Germany), 0.5% w/v L-ascorbic acid (Sigma-Aldrich, USA), 0.03% w/v proteinase K (Roche Diagnostics, Switzerland) and 4% 2-mercaptoethanol was added to the suspension. The lysis was carried out by incubation 45 min at 65°C. Isolated DNA was then purified by phenol-chloroform extraction. Finally, the DNA was precipitated with isopropanol and dissolved in sterile deionized water. RNA was eliminated from the samples by incubation 20 min at 37°C with 10 mg·l^-1^ ribonuclease A (Sigma-Aldrich, USA).

### Marker development and genotyping

The sequence assembly of *Bgh* isolate DH14 (available in the GenBank database under WGS project accession CAUH01000000; [33]) was used as template for marker development. Randomly selected sequence contigs exceeding 10 kb were considered for the analysis. First, the sequence contigs were aligned to a set of repetitive sequences specific for *B*. *graminis* available at database Repbase (http://www.girinst.org) [33,50] using BLASTN algorithm [51]. For one insertion site of each identified TE, two primer pairs were designed using software Primer3 (http://primer3.ut.ee) [52]. One pair was directed towards the identified LTR and the second was directed out of the LTR. In both cases, one primer of each pair was spanning the insertion site. The amplicon size was expected to range between 450–650 bp (S1 Table). RJMs were genotyped as presence/absence variation. An internal standard was used to verify that observed absence of PCR amplicon is not caused by a problem with PCR amplification. For this purpose, a 100 bp PCR fragment (Forward primer: ACGCACCCATGTTTGTCAT, Reverse primer: CCAATGGGGCAAGACAGTTA) of glyceraldehyde-3-phosphate dehydrogenase gene (GeneBank: X99732.1) was employed. Subsequently, SNP markers were derived from monomorphic RJM PCR products. RJMs which gave single PCR fragment for all tested samples were Sanger sequenced from both primers using BigDye^®^ Terminator v3.1 Cycle Sequencing Kit (Applied Biosystems, USA) and DNA Analyzer ABI 3730xl (Applied Biosystems, USA). Finally, SSR motifs were identified using a WebSat software (http://wsmartins.net/websat; [53]). To ensure sufficient resolution in native polyacrylamide gel electrophoresis and to increase the probability of finding polymorphism, only SSRs with unit length of 2–6 bp and comprising at least eight units were considered. Primers were designed in SSR flanking regions using the Primer3 software with expected amplicon size ranging from 70 to 150 bp (S3 Table).

### Genotyping of *Blumeria graminis* isolates

PCR amplification was carried out using C1000 Touch^™^ Thermal Cycler (Bio-Rad, USA) in 15 μl of reaction mix containing 10 mmol·l^-1^ Tris-HCl, 50 mmol·l^-1^ KCl, 1.5 mmol·l^-1^ MgCl_2_, 0.1% Triton X-100, 0.01% o-cresolsulfonephthalein, 1.5% sacharose, 0.2 mmol·l^-1^ of each dNTP (Fermentas, Lithuania), 0.3 U of Taq Polymerase (Fermentas, Lithuania), 1 μmol·l^-1^ of each forward and reverse primer and 500 pg of genomic DNA. The PCR conditions were as follows: 1) Initial denaturation at 95°C for 5 min; 2) 40 cycles comprising 30 s at 95°C, 30 s at 50°-60°C depending on particular primer pair, 1 min at 72°C; 3) Final extension at 72°C for 10 min. PCR products were separated using high-throughput Mega-Gel Vertical Electrophoresis system (C.B.S. Scientific, USA) on 4% (RJMs) or 6% (SSRs) non-denaturing polyacrylamide gels stained with ethidium bromide (Sigma-Aldrich, USA). All designed markers were tested using a selection of fifteen *B*. *graminis* isolates of the Czech Republic and the South African Republic (S7 Table). Markers polymorphic in this set were used for subsequent genotyping of all studied *Bgh* isolates.

### Data analysis

Polymorphic bands of SSR markers were scored separately as individual PAV markers. The same approach was applied for RJMs. MEGA5 software [54] was used for multiple alignment of DNA sequence data and identification of SNPs which were subsequently manually converted to binary data to match the output of PAV markers. Each SNP position was scored as individual marker. For SNPs with redundant genotypes (originating from different primer pairs designed for the same retrotransposon), only one marker was considered for further analysis. To demonstrate the power of the marker panel to discriminate individual *Bgh* isolates, a phylogenetic analysis was performed using software package PHYLIP 3.69 [55]. First, a set of binary data in the PHYLIP format was converted into distance matrix by Restdist tool. In the next step, the matrix was used for construction of an unrooted tree based on the neighbor-joining algorithm [56] by Neighbor tool selected due to its suitability for analyzing large datasets. Results were visualized by software Geneious 9.0.5 (Biomatters Ltd, Auckland, New Zealand). Population analysis of the isolate sets was carried out using software GenAlEx v6.502 [41]. All the analyses were performed with data loaded as binary (haploid). Polymorphism frequencies, diversity and genetic distance estimations were calculated using the tool “Frequency” with option Set-by-step. Binary genetic distance matrix was used for AMOVA analysis and for calculation of ΦPT (the analog of fixation index Fst when data are haploid or binary) with 999 permutations.

## Supporting Information

S1 Table Primer sequences and final markers based on retrotransposon insertion sites. (DOCX)

S2 Table Sequence-based markers derived from RJM amplicons. (DOCX)

S3 Table Primer sequences and final markers based on microsatellite loci. (DOCX)

S4 Table *Blumeria graminis* f. sp. *hordei* isolates used for marker validation. (DOCX)

S5 Table *Blumeria graminis* f. sp. *hordei* isolates collected across the Czech Republic in 2012. (DOCX)

S6 Table *Blumeria graminis* f. sp. *hordei* isolates collected in Australia in 2011. (DOCX)

S7 Table Worldwide collection of *Blumeria graminis* f. sp. *hordei* isolates provided by Agrotest Fyto Ltd. (DOCX)
