# Supplementary material for: Genetic Diversity of Blumeria graminis f. sp. hordei in Central Europe and Its Comparison with Australian Population
Source: PLoS One. 2016 Nov 22;11(11):e0167099. doi: 10.1371/journal.pone.0167099 (PMC5119828; doi:10.1371/journal.pone.0167099)
Supplement: S1 Table — (DOCX) [file pone.0167099.s001.docx]

**S1 Table.** Primer sequences and final markers based on retrotransposon insertion sites.

| **Marker** | **Primer sequences (5‘ – 3‘)** | **Expected amplicon size [bp]** | | **Polymorphism** | **DH14 contig** |
| --- | --- | --- | --- | --- | --- |
| *obm1* | GCATTGAAAGTGCTGGGAAT | | 533 | multiple amplicons | 000430 |
|  | GTTCTTGCCGGTGACTGATT | |  |  |  |
| *obm2* | CGCCATCTAGGCATAGAAAAA | | 609 | monomorphic |  |
|  | TTATATTCCCAGCACTTTCAATG | |  |  |  |
| *obm3* | CACAATCCAAAGATATGTTGAAGG | | 565 | monomorphic | 002314 |
|  | CTGCATCGAGACTTTCAGCA | |  |  |  |
| *obm4* | TCCAGTTGTGTTCTCTCTTCCTC | | 545 | monomorphic |  |
|  | TCATACCCTTCAACATATCTTTGG | |  |  |  |
| *obm5* | GAACTTGGAGAGCAGCCAAC | | 526 | no amplification | 001302 |
|  | CCTGTCAACACTTGGCGTGT | |  |  |  |
| *obm6* | GGAACCAACACCAAGACGAT | | 599 | SNP |  |
|  | ACCTGAACCCAATCCAGTCA | |  |  |  |
| *obm7* | CCATGATGTTGTGTTAGACACTTAG | | 562 | monomorphic | 007064 |
|  | CATCAAAATTGGCTTCGACA | |  |  |  |
| *obm8* | CTTCCATGGAGTTTTGGTTGA | | 525 | monomorphic |  |
|  | CCCTAAGTGTCTAACACAACATCA | |  |  |  |
| *obm9* | TGTCTTATCTTGTGTTGTGACTGG | | 537 | SNP^a^ | 003729 |
|  | TTGAGAGCTTGTGCATGTTTG | |  |  |  |
| *obm10* | CCCAGACATTTCCCTAGCTG | | 560 | SNP^a^ |  |
|  | TGCCAGTCACAACACAAGAT | |  |  |  |
| *obm11* | CAAGTATGCCTGGATGTAACAG | | 557 | no amplification | 003457 |
|  | AGCCCTAGTGAAGTGGGCTA | |  |  |  |
| *obm12* | CTAGCCGTCACACTGGATGA | | 579 | no amplification |  |
|  | GCGCCTACTCTGATGAGTCC | |  |  |  |
| *obm13* | AATTCTCCGCTATTGTGACG | | 525 | PAV^b^ | 001347 |
|  | TTCACCTTTCTGGCAAGTCA | |  |  |  |
| *obm14* | TCATTAAACTCTTACAGCTATGTCAAA | | 533 | PAV^b^ |  |
|  | CATGCCTTCATCATCCACTG | |  |  |  |
| *obm15* | GGGTGACGGTTGCCTAGTAA | | 470 | PAV^c^ | 002916 |
|  | CACCGTGACACGTGGTTATC | |  |  |  |
| *obm16* | TGCTCGATAACCACGTGTC | | 573 | PAV^c^ |  |
|  | GTAGCGGAAAGGGATGATGA | |  |  |  |
| *obm17* | CCGTGAATACCTGTGACAGAA | | 575 | monomorphic | 003563 |
|  | TTGATGTTGATGGCGAAGAA | |  |  |  |
| *obm18* | GGTCAAACCGTAACAATACCC | | 497 | PAV |  |
|  | TGGGCCTGATCACAACAGTA | |  |  |  |
| *obm19* | TTCCCTACTGTCAGGGCTCTC | | 534 | monomorphic | 007186 |
|  | TAAATCCCTCCGAACCGAAT | |  |  |  |
| *obm20* | AGTCCGTGACACCTACTCAGG | | 524 | SNP |  |
|  | TGAGGCTTGGGTAGAAGTCAA | |  |  |  |

^a, b^ Identical markers from the same insertions site

^c^ Unreliable genotyping
