# Supplementary material for: Genetic Diversity of Blumeria graminis f. sp. hordei in Central Europe and Its Comparison with Australian Population
Source: PLoS One. 2016 Nov 22;11(11):e0167099. doi: 10.1371/journal.pone.0167099 (PMC5119828; doi:10.1371/journal.pone.0167099)
Supplement: S2 Table — (DOCX) [file pone.0167099.s002.docx]

**S2 Table.** Sequence-based markers derived from RJM amplicons.

| **Marker** | **Polymorphism** | **Position in amplicon** | **Alleles** |
| --- | --- | --- | --- |
| *obm6* | *obm6.1* | 75 | A/G |
|  | *obm6.2* | 247 | C/A |
|  | *obm6.3* | 259 | T/C |
|  | *obm6.4* | 304 | G/A |
| *obm9* | *obm9.1* | 133 | T/C |
|  | *obm9.2* | 166 | 63 bp indel |
|  | *obm9.3* | 475 | A/T |
| *obm20* | *obm20.1* | 94 | T/A |
|  | *obm20.2* | 106 | A/G |
|  | *obm20.3* | 250 | C/T |
|  | *obm20.4* | 324 | G/T |
|  | *obm20.5* | 388 | A/T |
|  | *obm20.6* | 437 | T/A |
|  | *obm20.7* | 449 | C/T |
