# Supplementary material for: Genetic Diversity of Blumeria graminis f. sp. hordei in Central Europe and Its Comparison with Australian Population
Source: PLoS One. 2016 Nov 22;11(11):e0167099. doi: 10.1371/journal.pone.0167099 (PMC5119828; doi:10.1371/journal.pone.0167099)
Supplement: S3 Table — (DOCX) [file pone.0167099.s003.docx]

**S3 Table.** Primer sequences and final markers based on microsatellite loci.

| **Marker** | **Primer sequences (5‘ – 3‘)** | **SSR motif** | **Expected**  **amplicon size [bp]** | **Number of alleles** | **DH14 contig** |
| --- | --- | --- | --- | --- | --- |
| *obm21* | GTATGGTTTGAGGGGTGGTCTA | (CT)9 | 115 | - | 000403 |
|  | TTGACATTTTCTACTTCGCCCT |  |  |  |  |
| *obm22* | CCTAGTTTTGTGCACCAGCA | (GAAAAA)9 | 150 | -^a^ | 000935 |
|  | GGGTGAAACGGGCTGATAG |  |  |  |  |
| *obm23* | CGATCGCGTTTACTAGGTCA | (AT)19 | 108 | - | 001220 |
|  | TCCGGCACTGTAATGTGGTA |  |  |  |  |
| *obm24* | TCATGCCTCTTGGGTTTAGG | (ATAC)16 | 129 | 5 | 004927 |
|  | CTTGAAGCAAGCAATGGACA |  |  |  |  |
| *obm25* | CCAATTGGAACTCAACAAATG | (AT)11 | 88 | - | 006229 |
|  | AACTGAGTTAATGACTGTTGAGGA |  |  |  |  |
| *obm26* | TAGGCAGAGAGGACCTGCAT | (TAA)10 | 121 | -* | 002485 |
|  | CCTGTTCTGATCTGCACTCAA |  |  |  |  |
| *obm27* | AGCAATAGCATCTTCAGTTTTGA | (GT)9 | 97 | 3 | 003803 |
|  | CATGTCCTTACATAGTCGCAGA |  |  |  |  |
| *obm28* | GGCGTGACTGCGGTAACTAT | (CA)9 | 78 | 3 | 004468 |
|  | CGAGTCGAAGAACTGGATCG |  |  |  |  |
| *obm29* | GAGGAGCCCAATGTGCTAGA | (CACT)9 | 105 | 7 | 005093 |
|  | AGGATACAGGGAGGATTCAGG |  |  |  |  |
| *obm30* | TGGAGAGAGAATTCCTCAATCTAAA | (AT)10 | 98 | - | 005649 |
|  | TTCAGGTCACAGGGAGAATCTT |  |  |  |  |

^a^ Too complex patterns, irreproducible
