# Supplementary material for: Genetic Diversity of Blumeria graminis f. sp. hordei in Central Europe and Its Comparison with Australian Population
Source: PLoS One. 2016 Nov 22;11(11):e0167099. doi: 10.1371/journal.pone.0167099 (PMC5119828; doi:10.1371/journal.pone.0167099)
Supplement: S4 Table — (DOCX) [file pone.0167099.s004.docx]

**S4 Table.** *Blumeria graminis* f. sp. *hordei* isolates used for marker validation.

| **Pathotype** | **Isolate designation** | **Locality of collection** | **Date of collection** |
| --- | --- | --- | --- |
| 3775 | K200/2009 | Praha – Ejpovice^a^ | 4.6.2009 |
| 4517 | B45/2009 | Brno – Břeclav^a^ | 28.5.2009 |
| 4553 | O283/2009 | Praha – Chlumec n. Cidlinou^a^ | 4.6.2009 |
| 5725 | M232/2009 | Praha – Lovosice^a^ | 4.6.2009 |
| 5771 | K190/2009 | Praha – Ejpovice^a^ | 4.6.2009 |
| 5774 | I167/2009 | Gas station Avanti – Praha^a^ | 4.6.2009 |
| 7455 | M236/2009 | Praha – Lovosice^a^ | 4.6.2009 |
| 0020^b^ | 65/2004 | South African Republic (Hartswater) | 23.9.2004 |
| 0422^b^ | 4/20/2007 | South African Republic (Caledon) | 23.9.2007 |
| 1765 | I162/2009 | Gas station Avanti – Praha^a^ | 4.6.2009 |
| 4114 | I158/2009 | Gas station Avanti – Praha^a^ | 4.6.2009 |
| 4773 | A23/2009 | Kroměříž – Brno^a^ | 28.5.2009 |
| 5775 | L209/2009 | Praha – Řevničov^a^ | 4.6.2009 |
| 7555 | M246/2009 | Praha – Lovosice^a^ | 4.6.2009 |
|  | Tm258 | Olomouc | 2010 |

The isolates were collected in the Czech Republic, if not indicated otherwise.

^a^ Quoted municipalities or gas stations are delimiting passages of Czech highways

^b^ Included in core collection of *Bgh* isolates maintained at Agrotest Fyto Ltd.
