# Supplementary material for: Genetic Diversity of Blumeria graminis f. sp. hordei in Central Europe and Its Comparison with Australian Population
Source: PLoS One. 2016 Nov 22;11(11):e0167099. doi: 10.1371/journal.pone.0167099 (PMC5119828; doi:10.1371/journal.pone.0167099)
Supplement: S5 Table — (DOCX) [file pone.0167099.s005.docx]

**S5 Table.** *Blumeria graminis* f. sp. *hordei* isolates collected across the Czech Republic in 2012.

| **Isolate designation^b^** | **Locality of collection^a^** | **Date of collection** |
| --- | --- | --- |
| A1, A2, A3, A4, A5, A6, A7, A8, A9, A10, A11, A12 | Kromeříž – Brno | 26.5.2012 |
| B1, B2, B3, B4, B5, B6 | Brno – Lechovice | 25.5.2012 |
| C1, C2, C3, C4, C5, C6, C7, C8, C9, C10, C11, C12 | Brno – Břeclav | 25.5.2012 |
| D1, D2, D3, D4, D5 | Vyškov – Olomouc – Velká Bystřice | 24.5.2012 |
| E1, E2, E3, E4, E5, E6, E9, E13, E14, E16, E19, E20, E25, E34, E37 | Velká Bystřice – Ostrava Vítkovice | 24.5.2012 |
| F1, F2, F3 | Olomouc – Zábřeh na Moravě | 24.5.2012 |
| I2, I4, I11, I16, I21, I24, I25, I26, I27, I28, I30, I31, I32, I33, I36 | D1 highway km 90 – Praha Stodůlky | 26.5.2012 |
| L1, L2 | Praha Stodůlky – Karlovy Vary | 26.5.2012 |
| M1, M2, M3, M4, M5, M6 | Praha – Lovosice | 25.5.2012 |
| N1, N2, N3, N5 | Praha – Mladá Boleslav – Březina | 25.5.2012 |
| O1, O2, O3 | Praha – Hradec Králové | 25.5.2012 |
| X1, X3, X5, X7, X10, X11, X15, X16, X19, X24, X25, X26, X28, X34 | Brno - D1 highway km 90 | 26.5.2012 |

^a^ Quoted municipalities are delimiting passages of highway

^b^ Letters included in the names of isolates corresponds to regions illustrated on Figure 1.
