# Supplementary material for: Genetic Diversity of Blumeria graminis f. sp. hordei in Central Europe and Its Comparison with Australian Population
Source: PLoS One. 2016 Nov 22;11(11):e0167099. doi: 10.1371/journal.pone.0167099 (PMC5119828; doi:10.1371/journal.pone.0167099)
Supplement: S6 Table — (DOCX) [file pone.0167099.s006.docx]

**S6 Table.** *Blumeria graminis* f. sp. *hordei* isolates collected in Australia in 2011.

| **Isolate designation** | **Locality of collection** | **Date of collection** |
| --- | --- | --- |
| 501, 504, 506, 537, 539, 654 | Toowoomba (Queensland) | 7.9.2011 |
| 507, 508, 512, 514, 544, 548, 550, 575, 576, 655 | Tamworth (New South Wales) | 15.9.2011 |
| 518, 519, 520, 521, 522, 553 | Rosedale (South Australia) | 22.9.2011 |
| 534, 559, 561, 566, 569 | Horsham (Victoria) | 14.9.2011 |
| 541, 693 | Gatton (Queensland) | 7.9.2011 |
| 591, 594, 600, 605, 606, 607, 692 | Field (Tasmania) | 5.10.2011 |
| 616, 618, 620, 643, 648 | Perth (Western Australia) | 5.10.2011 |
| 680, 682, 683, 684, 685, 686, 687, 689, 690 | Wagga Wagga (New South Wales) | 13.10.2011 |
