# Supplementary material for: Genetic Diversity of Blumeria graminis f. sp. hordei in Central Europe and Its Comparison with Australian Population
Source: PLoS One. 2016 Nov 22;11(11):e0167099. doi: 10.1371/journal.pone.0167099 (PMC5119828; doi:10.1371/journal.pone.0167099)
Supplement: S7 Table — (DOCX) [file pone.0167099.s007.docx]

**S7 Table.** Worldwide collection of *Blumeria graminis* f. sp. *hordei* isolates provided by Agrotest Fyto Ltd.

| **Isolate designation** | **Origin^a^** | **Date of collection** |
| --- | --- | --- |
| 3-33/2003 | Yancheng, China (CHN) | 13.5.2003 |
| 65/2004 | South African Republic (ZAF) | 23.9.2004 |
| 655/2011 | Tamworth, New South Wales, Australia (AUS) | 15.9.2011 |
| 290/2010 | Breeza, New South Wales, Australia (AUS) | 16.9.2010 |
| 54/2005 | Bizcocho, Uruguay (URY) | 7.10.2005 |
| 4/20/2007 | South African Republic (ZAF) | 23.9.2007 |
| Race I | Japan, introduction from Denmark (JPN) | 1953 |
| 63-1/1997 | Kroměříž, Czech Republic (CZE) | 6.6.1997 |
| X30/2012 | D1 highway km 95 – Brno, Czech Republic (CZE) | 26.5.2012 |
| H-148 | Israel (ISR) | 1979 |
| Y-069 | Israel (ISR) | 1979 |

^a^ Three-letter codes in parentheses corresponding to the country of origin are used on Figure 2
